# Up-regulation of *NCED3* and ABA biosynthesis occur within minutes of a decrease in leaf turgor but *AHK1* is not required

**DOI:** 10.1093/jxb/erx124

**Published:** 2017-04-25

**Authors:** Frances C Sussmilch, Timothy J Brodribb, Scott A M McAdam

**Affiliations:** School of Biological Sciences, University of Tasmania, Hobart, TAS, Australia

**Keywords:** ABA, AHK1, leaf turgor, NCED, stomata, turgor sensor, VPD

## Abstract

A major environmental signal influencing day-time stomatal aperture is the vapour pressure deficit between the leaf and atmosphere (VPD). In angiosperms, increased VPD triggers biosynthesis of abscisic acid (ABA), prompting rapid stomatal closure. Altered cell turgor has been proposed as the trigger for ABA biosynthesis, but the timing and nature of the genetic signals linking these processes have remained uncertain. We investigated this in Arabidopsis by examining changes induced by a decrease in leaf turgor, simulating a natural increase in VPD. We found that the rate-limiting gene within the *de novo* ABA biosynthesis pathway, 9-*cis*-epoxycarotenoid dioxygenase 3 (*NCED3*), was induced and ABA levels increased within just 5 min of decreased leaf turgor. This rapid induction matches the time-frame for initiation of stomatal closure in response to a doubling in VPD. We further examined Arabidopsis histidine kinase1 (AHK1) as the most likely candidate for the turgor-sensing receptor involved, but found no significant difference between wild-type and an *ahk1* null mutant in the induction of ABA-biosynthetic genes, ABA production, or stomatal behaviour. We show that decreased leaf turgor triggers *de novo* ABA biosynthesis within the time-frame of the stomatal response to VPD, but that AHK1 does not fulfil a critical role as a turgor-sensing receptor within this pathway.

## Teaser

Decreased leaf turgor triggers up-regulation of the rate-limiting *NCED3* gene for *de novo* biosynthesis of ABA within the narrow time-frame required to initiate stomatal closure at increased vapour pressure deficit.

## Introduction

A major advancement that allowed plants to successfully colonise dry terrestrial environments was the evolution of stomata as a means of controlling plant water balance during photosynthesis (Raven, 2002). The majority of water loss in land plants occurs by transpiration through open stomata during photosynthesis. Angiosperms have evolved the ability to integrate multiple environmental signals for the precise regulation of stomatal aperture, fine-tuning the balance between maximising photosynthesis and avoiding damaging dehydration (Cowan and Farquhar, 1977; Brodribb and Holbrook, 2003). One of the most important environmental signals to influence stomatal aperture throughout the day is a change in the humidity of the air, or more precisely, the vapour pressure deficit between the leaf and the atmosphere (VPD). The phytohormone abscisic acid (ABA) plays a central role in the closure of angiosperm stomata in response to high VPD (Xie *et al.*, 2006). An increase in ABA level prompts the activation of ion channels in guard cells that actively decrease their osmotic potential, causing the guard cells to lose turgor and close the stomatal pore (Keller *et al.*, 1989; Geiger *et al.*, 2009). An increasing body of evidence suggests that ABA biosynthesis at high VPD is essential for normal stomatal closure in angiosperms (Bauerle *et al.*, 2004; Xie *et al.*, 2006; Bauer *et al.*, 2013; McAdam and Brodribb, 2015; McAdam *et al.*, 2016). Studies have shown that (i) a sufficient increase in VPD triggers the biosynthesis of ABA (Bauer *et al.*, 2013; McAdam and Brodribb, 2016), and (ii) by the time stomata have responded to increased VPD (within 20 min), only a single gene encoding the rate-limiting enzyme 9-*cis*-epoxycarotenoid dioxygenase, *NCED*, is significantly up-regulated within the ABA biosynthesis pathway (Qin and Zeevaart, 1999; Thompson *et al.*, 2000; McAdam *et al.*, 2016). However, the stomatal response to VPD can occur over an extremely rapid time-frame, with incipient stomatal closure at increased VPD recorded within a minute following the change in VPD (Fanjul and Jones, 1982). The rapid speed of the stomatal response to VPD has led a number of authors to propose other mechanisms as alternatives to *de novo* ABA biosynthesis, to explain the increase in ABA level driving these stomatal responses (Lee *et al.*, 2006; Georgopoulou and Milborrow, 2012). Precise, time-resolved information about the speed at which *NCED* transcription can be induced and ABA levels can increase, following a change in VPD, would resolve this debate. Even the nature of the signal by which plants sense changes to VPD remains to be explained. Candidates such as leaf turgor and osmotic potential vary in proportion to transpiration rate and have been raised as possibilities, while even direct sensing of atmospheric humidity has been proposed (Buckley, 2016).

A recent study using a novel method of modifying cell turgor by the application of external pressure indicates that a decline in cell turgor is likely to be the physiological trigger for induction of ABA biosynthesis when leaves are exposed to high VPD in angiosperms (McAdam and Brodribb, 2016). This suggests the existence of a molecular signalling pathway linking the perception of pressure by a turgor-sensing protein to the up-regulation of *NCED* expression and increase in ABA level at high VPD. However, this key molecular component remains as yet unidentified. Results from a series of studies suggest that the transmembrane protein Arabidopsis histidine kinase1 (AHK1) is the most likely candidate for the turgor-sensing receptor within this pathway. AHK1 is a homolog of the yeast protein synthetic lethal of N-end rule1 (SLN1), which acts as a receptor within the osmotic stress signalling pathway and alters expression of downstream stress-response genes via a response regulator and mitogen-activated protein kinase (MAPK) signalling cascade (Urao *et al.*, 1999). SLN1 is usually referred to as an osmosensor; however, the signal actually perceived is thought instead to be changes in either turgor pressure (Reiser *et al.*, 2003), or cell wall structure (Shankarnarayan *et al.*, 2008). In *Arabidopsis thaliana*, AHK1 is a positive regulator of drought and osmotic stress responses, and is involved in ABA signalling (Urao *et al.*, 1999; Tran *et al.*, 2007). *NCED3* and numerous other genes within the ABA-biosynthesis pathway have been found to have reduced expression in an *ahk1* null mutant background and increased expression in a line over-expressing *AHK1* when plants are exposed to high osmolarity over a 10–15 h period (Wohlbach *et al.*, 2008). However, there are conflicting reports over whether this difference in expression results in a significant difference in ABA levels in these plants (Wohlbach *et al.*, 2008; Kumar *et al.*, 2013). While the importance of *AHK1* for sensing changes in water potential over a longer time-frame have been questioned (Kumar *et al.*, 2013), this protein provides the best candidate yet for the hypothesised turgor-sensing receptor that rapidly up-regulates ABA-biosynthesis when plants are exposed to increased VPD.

In this study, we address two key questions: (i) does a controlled reduction in leaf turgor trigger increased expression of *NCED3* over the short time-frame of the stomatal response to VPD, and (ii) is AHK1 the turgor-sensing receptor responsible for up-regulating ABA-biosynthesis during exposure to high VPD?

## Materials and methods

### Plant materials and growth conditions

Wild-type *Arabidopsis thaliana* Wassilewskija (Ws-2) and null mutant *ahk1-4* (Wohlbach *et al.*, 2008) were grown under the conditions described by McAdam and Brodribb (2016). The turgor of recently excised leaves was reduced by the controlled method of pressurisation in a Scholander pressure chamber and gas exchange measurements of stomatal responses to VPD were undertaken as described by McAdam and Brodribb (2016). In this study, we applied 1.5 MPa of external pressure to excised leaves for 1, 5, 10 or 20 min, after which tissue samples for gene expression analysis were snap-frozen in liquid nitrogen. Leaf samples were taken for the quantification of ABA after 5, 10 and 20 min for wild-type, and after 20 min only for *ahk1-4*. Initial samples for gene expression and ABA quantification were taken prior to application of external pressure for both genotypes, while controls for ABA level comprised leaves that were contained in the pressure chamber without external pressure for 5, 10 and 20 min. The pressure level of 1.5 MPa was chosen as it is known to be beyond the threshold trigger point for ABA biosynthesis in this genotype of Arabidopsis (McAdam and Brodribb 2016).

### Measurement of ABA levels

Foliar ABA quantification was performed using the method of McAdam (2015). For statistical analysis of ABA levels, one-way ANOVA was conducted with Tukey’s HSD *post-hoc* test to compare initial and final (20 min) levels between wild-type and *athk1-4*, and with Dunnett two-sided *t*-tests to compare 5 and 10 min levels with initial levels in the wild-type.

### Expression analyses

For expression experiments, RNA was extracted using the Agilent Plant RNA Isolation Mini Kit according to the manufacturer’s instructions, and RNA quantification, reverse transcription, and quantitative reverse transcription PCR (qRT-PCR) were conducted as previously described (McAdam *et al.*, 2016). Transcript levels for each gene of interest were evaluated for 2–4 replicates per genotype/time-point against the SAND family gene *MONENSIN SENSITIVITY1* (*MON1*; At2g28390) using the primers of Czechowski *et al.* (2005), as this reference gene was found to be stably expressed in these samples. Primers were as follows: *CYP707A1*, 5′-AACTCAGGAAGCTTGTTCTTCG-3′ and 5′-AGATCGATAGCAACGCAACG-3′; *CYP707A3*, 5′-AAGC AGGATTAACCGACGAAC-3′ and 5′-ATTGCCATTTGCTCTTC AGTG-3′. Primers for all other genes are as previously described (McAdam *et al.*, 2016).

For calculations of relative gene expression, the comparative *C*_T_ method was used to determine the difference in threshold cycle between the gene of interest and *MON1* (∆*C*_T_) for each sample, and the fold-change between control (initial samples taken prior to treatment) and other sample groups (2^−∆∆*C*T^), as previously described (Schmittgen and Livak, 2008). Statistical analysis of expression data was conducted on ∆*C*_T_ values for each sample, using one-way ANOVA with either Tukey’s HSD *post-hoc* test for comparisons of initial and final levels between genotypes, or Dunnett two-sided *t*-tests for comparisons of multiple time-points with initial values within a genotype, with a significance level of *P*<0.05 unless otherwise stated. All statistical analyses were conducted using IBM SPSS Statistics (version 21).

## Results and Discussion

### The speed of induction of ABA biosynthesis via NCED3

**Fig. 1.**
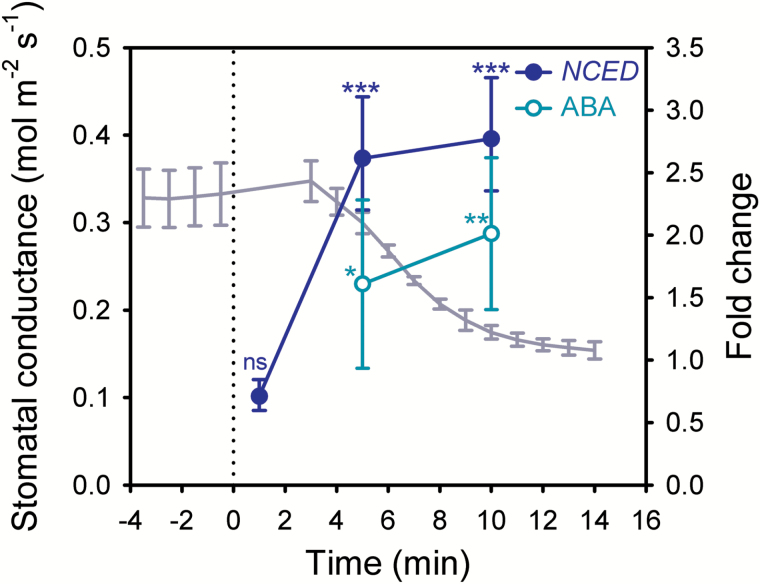
*NCED3* expression and ABA levels increase after a decrease in leaf turgor and correspond with the timing of stomatal closure after a step-increase in VPD in wild-type Arabidopsis. The mean response of stomatal conductance to a step-change in VPD from 0.75 to 1.5 kPa (line with no symbols; *n*=3, ±SE) and the fold-change in *NCED3* expression (closed circles; *n*=3, ±95% CI) and foliar ABA levels (open circles; *n*=3, ±95% CI) after exposure to external pressure of 1.5 MPa, relative to initial levels. The timing of initiation of the change in external pressure or VPD is denoted by a vertical dotted line. Asterisks denote significant changes compared to initial levels: *P≤0.05; **P≤0.01; ***P≤0.001; ns, not significant. (This figure is available in colour at *JXB* online.)

We first investigated wild-type expression of *NCED3*, the gene encoding the key, rate-limiting enzyme for ABA-biosynthesis (Qin and Zeevaart, 1999; Thompson *et al.*, 2000). We examined *NCED3* induction over a short time-course of 1–10 min after exposure of leaves to an external pressure of 1.5 MPa, a method that specifically reduces leaf turgor similar to the effect of a natural increase in VPD (McAdam and Brodribb, 2016). Relative to initial levels, expression of *NCED3* was not significantly different after 1 min of exposure to external pressure (*P*=0.185), but a significant doubling of *NCED3* levels occurred within 5 min of exposure (*P*=0.001; Fig. 1). We also detected a functionally significant increase in foliar ABA level within 5 min of altered leaf turgor pressure (*P*=0.047), which was more pronounced after 10 min of exposure (*P*=0.005; Fig. 1). The time-frame for detected increases in *NCED3* and ABA levels coincided with the initiation of stomatal closure in response to a doubling in VPD (Fig. 1) (Xie *et al.*, 2006), providing evidence that *de novo* ABA biosynthesis, triggered by reduced leaf turgor, can occur within this narrow time-frame.

We also examined the genes that encode the first enzyme in the major ABA catabolism pathway, ABA 8′-hydroxylase, in guard cells and vascular tissue, *CYP707A1* and *CYP707A3*, respectively (Kushiro *et al.*, 2004; Okamoto *et al.*, 2009). We found *CYP707A3* to be significantly upregulated within 5 min of altered leaf turgor pressure (*P*=0.0003), while *CYP707A1* was significantly up-regulated after 10 min of exposure (*P*=0.0004), relative to initial levels (Supplementary Fig. S1 at *JXB* online). These results are consistent with previous findings that show these genes to be up-regulated by drought stress, a response that is at least partially ABA-dependent (Kushiro *et al.*, 2004; Saito *et al.*, 2004; Umezawa *et al.*, 2006). Our results confirm there is no down-regulation in catabolism contributing to the rapid increase in ABA levels observed in response to altered leaf turgor, and instead support the conclusion that this rapid increase in ABA levels is due to an increase in ABA biosynthesis, via rapid *NCED3* up-regulation.

For further confirmation that the application of external pressure was the trigger for *NCED3* induction in our short time-course experiment, we conducted additional replicate experiments. Firstly, we showed that no significant change in *NCED3* expression was induced in wild-type leaves in the pressure chamber when no pressure was applied, even after up to 20 min (*P*=0.688; Supplementary Fig. S2), nor were ABA levels changed in wild-type leaves at any time point when no pressure was applied (*P*=0.911; Supplementary Fig. S3). Secondly, as our methods of external pressure application in air could potentially affect carbon dioxide (CO_2_) availability, and increased CO_2_ levels can trigger stomatal closure (Mott, 1988), we ruled out the possibility that the induction of *NCED3* we detected may have been due to altered CO_2_ levels, by repeating this experiment under compressed nitrogen gas. We found that *NCED3* was similarly induced by application of external pressure for 20 min when performed under nitrogen as when performed under air (Supplementary Fig. S4). The results from these combined experiments provide evidence that *de novo* biosynthesis of ABA is up-regulated in response to decreased leaf turgor via the rapid induction of *NCED3*, which can occur within a time-frame that matches the speed of the stomatal response to VPD in Arabidopsis.

### Investigating AHK1 as a candidate turgor-sensing receptor

**Fig. 2.**
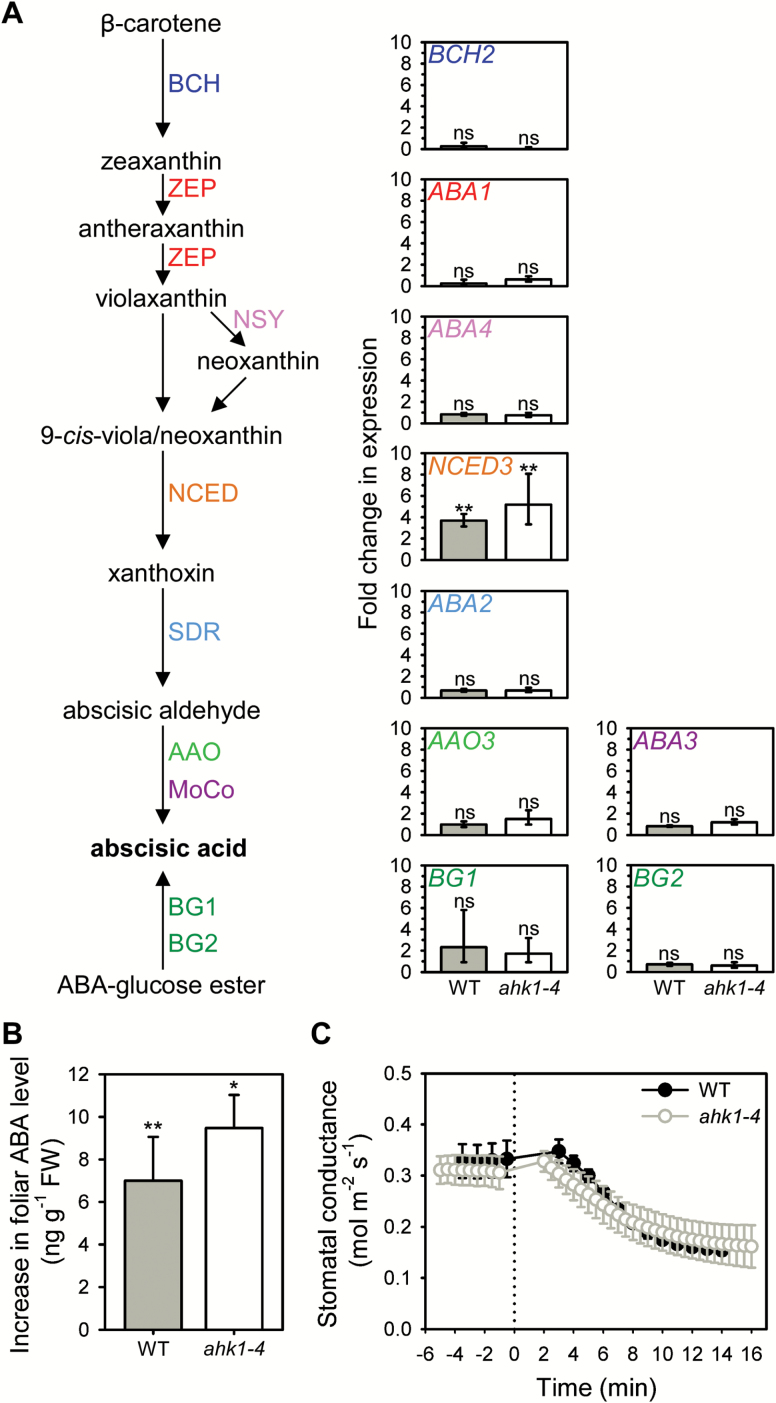
Loss of *AHK1* function does not affect the expression of ABA biosynthesis genes or ABA level after a decrease in leaf cell turgor, or the stomatal response to increasing VPD. (A) The ABA biosynthesis and ABA-glucose ester hydrolysis pathways showing the fold-change in relative expression of the key genes involved (±95% CI, *n*=2–3), and (B) the mean increase in foliar ABA level (±95% CI, *n*=3), after 20 min exposure to external pressure of 1.5 MPa, relative to initial values. Asterisks denote significant changes compared to initial levels within each genotype: *P≤0.05; **P≤0.01; ns, not significant. (C) Mean response of stomatal conductance to a step-change in VPD from 0.75 to 1.5 kPa [*n*=3, ±SE; dotted line denotes time of VPD change; wild-type (WT) data is also shown in Fig. 1]. (This figure is available in colour at *JXB* online.)

We next examined AHK1 as a possible candidate for the turgor-sensing receptor acting upstream of *NCED* in this VPD-response pathway, using the null *ahk1-4* mutant (Wohlbach *et al.*, 2008). In wild-type Arabidopsis, consistent with previous results following exposure to increased VPD (McAdam *et al.*, 2016), we found that *NCED3* was the only gene within the ABA biosynthetic or conjugation pathway to show a significant change in expression within 20 min of exposure of excised leaves to an external pressure of 1.5 MPa (Fig. 2A). However, we detected no significant difference between the wild-type and *ahk1-4* mutant in either initial (*P*=0.058) or final (*P*=0.387) levels of *NCED3* expression, and both genotypes showed a similar pressure-induced increase in *NCED3* expression (Fig. 2A). Accordingly, we found no significant difference in ABA levels between wild-type and *ahk1-4* before (*P*=0.421) or after 20 min of external pressure (*P*=0.994), and both genotypes also had a similar and significant pressure-induced increase in ABA level (Fig. 2B). In addition, we found that the stomatal response to a step-increase in VPD was not altered in the absence of AHK1 function (Fig. 2C). These results show that AHK1 does not function as an essential turgor-sensing receptor in the pathway for ABA-mediated stomatal closure in response to increased VPD.

The question of which turgor receptor may be fulfilling this critical role in angiosperms still remains. Based on knowledge of the molecular components involved in osmosensing in the simple eukaryote yeast, a number of other possible candidates have been suggested, including other histidine kinases closely related to AHK1, receptor-like kinases (RLKs; ~600 in Arabidopsis), and integrin-like proteins, which remain to be investigated for a role in regulating ABA biosynthesis (Christmann *et al.*, 2013; Osakabe *et al.*, 2014). Also, although it is likely that a single turgor-sensing protein first evolved to regulate ABA biosynthesis, as the simplest evolutionary model, it remains a possibility that a number of proteins may have since evolved some level of redundancy in this role.

**Fig. 3.**
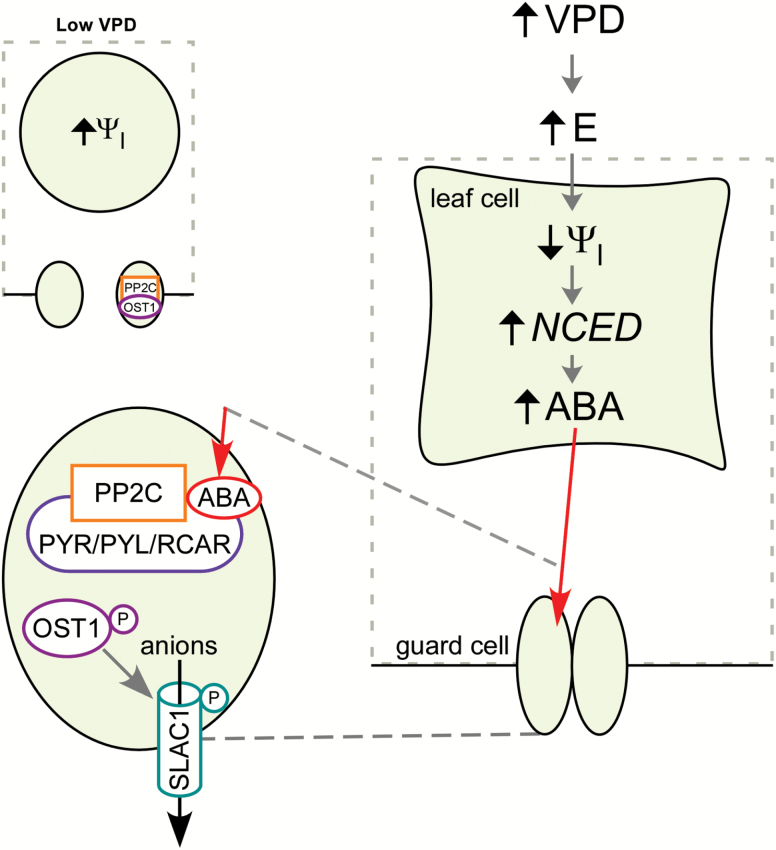
A proposed mechanistic model for ABA-mediated stomatal closure in response to increased VPD in angiosperms. At low VPD (left), leaf water potential (ψ _l_) and turgor pressure are high, and PROTEIN PHOSPHATASE TYPE 2C (PP2C) proteins inhibit the function of the key ABA-signalling pathway, the protein kinase OPEN STOMATA1 (OST1) (Ng *et al.*, 2011). This leaves OST1 incapable of activating the anion channel SLOW ANION CHANNEL 1 (SLAC1) in the guard cell membrane (Geiger *et al.*, 2009). When VPD increases (right), transpiration (E) increases, decreasing leaf water potential (ψ _l_) and leaf turgor pressure. Our results indicate that altered turgor is the likely trigger for rapid up-regulation of *NCED3*, the rate-limiting step in the ABA-biosynthesis pathway, resulting in an increase in ABA level within 5 min. The speed of this response indicates that the turgor sensor involved is located close to the stomata, and we hypothesise that it is in the mesophyll cells. ABA is transported into the guard cells (enhanced, centre), from the main site of synthesis in the leaf, where it binds with ABA receptors within the PYRABACTIN RESISTANCE 1 (PYR)/PYR1-LIKE (PYL)/REGULATORY COMPONENT OF ABA RECEPTOR (RCAR) family, which alleviate inhibition of OST1 by binding the catalytic site of PP2C proteins (Park *et al.*, 2009; Soon *et al.*, 2012). OST1 phosphorylates and thereby activates SLAC1, resulting in a flow of ions and loss of osmotic potential from the guard cells, which deflate, closing the stomatal pore (Chen *et al.*, 2010). (This figure is available in colour at *JXB* online.)

Here, we have shown that specifically altering leaf turgor through the application of external pressure is sufficient to trigger ABA biosynthesis through the up-regulation of *NCED3* within the rapid time-frame of the stomatal response to VPD. This adds further support to the argument that altered turgor constitutes the signal by which angiosperms detect day-time variation in VPD, in order to adjust stomatal aperture accordingly. We have summarised these findings in an updated mechanistic model for ABA-mediated stomatal closure in response to increased VPD in angiosperms (Fig. 3). While our results do not focus on the specific location of ABA-biosynthesis within the leaf, the speed of the response strongly suggests that the site of synthesis, and therefore the location of the turgor sensor that triggers *NCED3* up-regulation, is relatively close to the stomata, in the transpiration stream. All genes involved in ABA biosynthesis are expressed within guard cells, indicating that ABA biosynthesis can occur within guard cells (Bauer *et al.*, 2013). However, a number of lines of evidence suggest that ABA derived from either leaf vascular or mesophyll tissue that is transported into guard cells plays the predominant role in driving stomatal closure at high VPD (Fig. 3). Firstly, *NCED3* and genes that control the subsequent steps in the ABA biosynthetic pathway, *ABA2* and *AAO3*, are expressed predominantly in leaf vascular tissues (Cheng *et al.*, 2002; Koiwai *et al.*, 2004; Endo *et al.*, 2008). Secondly, mutants that lack the function of ABA uptake transporters in the guard cell membrane wilt faster than wild-type plants in response to drought stress (Kang *et al.*, 2010). Lastly, as ABA synthesis must precede loss of guard cell turgor for ABA-driven stomatal closure, in order for the VPD response to be controlled by guard-cell autonomous synthesis of ABA at high VPD there would need to be an as yet unidentified, turgor-independent trigger for ABA biosynthesis that directly senses humidity changes, an arguably less likely scenario (Grantz, 1990). It is worth noting that a recent study has shown that extreme mechanical changes occur in response to transpiration driven decreases in leaf water potential, with considerable yet reversible collapse of minor veins occurring within 20 s following a decrease in water potential in angiosperm leaves (Zhang *et al.*, 2016). This mechanical deformation of the xylem conduits would place mechanical stress on the surrounding cells and may contribute as a mechanical trigger in addition to altered turgor for up-regulation of ABA; a possibility that remains to be investigated.

## Conclusions

In conclusion, our results show that *de novo* biosynthesis of ABA, via up-regulation of the rate-limiting *NCED3* gene, can be triggered by changes in leaf turgor over the extremely fast time-frame required to initiate a stomatal response to VPD. These stomatal responses to VPD, governed by *NCED3*, are some of the most important determinants of diurnal gas exchange in land plants (Zhao and Running, 2010). As a result, the regulation of this gene has arguably one of the greatest influences on terrestrial productivity and transpiration of any signalling pathway in land plants. We find that a loss of function of the transmembrane protein AHK1 does not affect either the induction of ABA biosynthesis in response to altered leaf turgor, or stomatal closure in response to increased VPD. These results indicate that AHK1 does not function as the critical turgor-sensing receptor in the pathway for ABA-mediated stomatal closure in response to increased VPD. Instead, we suggest that the continued search for the main turgor receptor in this pathway should focus on the vascular tissue, which could be the predominant site of ABA synthesis (Endo *et al.*, 2008). Future characterisation of this important turgor-sensing receptor will be essential for shedding much needed light on this central means for regulating day-time transpiration in angiosperms.

## Supplementary data

Supplementary data are available at *JXB* online.

Fig. S1. Expression of ABA catabolism genes.

Fig. S2. Confirmation that *NCED3* up-regulation is triggered by application of external pressure.

Fig. S3. Confirmation that foliar ABA levels do not increase without the application of external pressure.

Fig. S4. Confirmation that *NCED3* up-regulation is triggered by changes to turgor pressure and not altered CO_2_ levels when external pressure is applied.

## Author contributions

SM conceived and designed the research and TB provided expertise. FS and SM conducted the experiments and analysed the data. FS wrote the manuscript. All authors read and approved the manuscript.

## Supplementary Material

supplementary_figures_S1_S4

## Abbreviations:

ABA: abscisic acid
AHK1: Arabidopsis histidine kinase1
NCED: 9-*cis*-epoxycarotenoid dioxygenase
qRT-PCR: quantitative reverse transcription PCR
SLN1: synthetic lethal of N-end rule1
VPD: vapour pressure deficit.

## References

[CIT0001] Bauer H Ache P Lautner S 2013 The stomatal response to reduced relative humidity requires guard cell-autonomous ABA synthesis. Current Biology 23, 53–57. 23219726 10.1016/j.cub.2012.11.022

[CIT0002] Bauerle WL Whitlow TH Setter TL Vermeylen FM 2004 Abscisic acid synthesis in *Acer rubrum* L. leaves—a vapor-pressure-deficit-mediated response. Journal of the American Society for Horticultural Science 129, 182–187.

[CIT0003] Brodribb TJ Holbrook NM 2003 Stomatal closure during leaf dehydration, correlation with other leaf physiological traits. Plant Physiology 132, 2166–2173. 12913171 10.1104/pp.103.023879 PMC181300

[CIT0004] Buckley TN 2016 Stomatal responses to humidity: has the ‘black box’ finally been opened?Plant, Cell & Environment 39, 482–484. 10.1111/pce.12651 26485479

[CIT0005] Chen YH Hu L Punta M Bruni R Hillerich B Kloss B Rost B Love J Siegelbaum SA Hendrickson WA 2010 Homologue structure of the SLAC1 anion channel for closing stomata in leaves. Nature 467, 1074–1080. 20981093 10.1038/nature09487 PMC3548404

[CIT0006] Cheng WH Endo A Zhou L 2002 A unique short-chain dehydrogenase/reductase in Arabidopsis glucose signaling and abscisic acid biosynthesis and functions. The Plant Cell 14, 2723–2743. 12417697 10.1105/tpc.006494 PMC152723

[CIT0007] Christmann A Grill E Huang J 2013 Hydraulic signals in long-distance signaling. Current Opinion in Plant Biology 16, 293–300. 23545219 10.1016/j.pbi.2013.02.011

[CIT0008] Cowan IR Farquhar GD 1977 Stomatal function in relation to leaf metabolism and environment. Symposia of the Society for Experimental Biology 31, 471–505. 756635

[CIT0009] Czechowski T Stitt M Altmann T Udvardi MK Scheible WR 2005 Genome-wide identification and testing of superior reference genes for transcript normalization in Arabidopsis. Plant Physiology 139, 5–17. 16166256 10.1104/pp.105.063743 PMC1203353

[CIT0010] Endo A Sawada Y Takahashi H 2008 Drought induction of Arabidopsis 9-*cis*-epoxycarotenoid dioxygenase occurs in vascular parenchyma cells. Plant Physiology 147, 1984–1993. 18550687 10.1104/pp.108.116632 PMC2492653

[CIT0011] Fanjul L Jones HG 1982 Rapid stomatal responses to humidity. Planta 154, 135–138. 24275973 10.1007/BF00387906

[CIT0012] Geiger D Scherzer S Mumm P Stange A Marten I Bauer H Ache P Matschi S Liese A Al-Rasheid KA 2009 Activity of guard cell anion channel SLAC1 is controlled by drought-stress signaling kinase-phosphatase pair. Proceedings of the National Academy of Sciences, USA 106, 21425–21430. 10.1073/pnas.0912021106 PMC2795561 19955405

[CIT0013] Georgopoulou Z Milborrow BV 2012 Initiation of the synthesis of ‘stress’ ABA by (+)-[^2^H_6_]ABA infiltrated into leaves of *Commelina communis*. Physiologia Plantarum 146, 149–159. 22471592 10.1111/j.1399-3054.2012.01630.x

[CIT0014] Grantz DA 1990 Plant response to atmospheric humidity. Plant, Cell & Environment 13, 667–679.

[CIT0015] Kang J Hwang J-U Lee M Kim Y-Y Assmann SM Martinoia E Lee Y 2010 PDR-type ABC transporter mediates cellular uptake of the phytohormone abscisic acid. Proceedings of the National Academy of Sciences, USA 107, 2355–2360. 10.1073/pnas.0909222107 PMC2836657 20133880

[CIT0016] Keller BU Hedrich R Raschke K 1989 Voltage-dependent anion channels in the plasma membrane of guard cells. Nature 341, 450–453. 10.1002/j.1460-2075.1990.tb07608.x PMC552158 1701140

[CIT0017] Koiwai H Nakaminami K Seo M Mitsuhashi W Toyomasu T Koshiba T 2004 Tissue-specific localization of an abscisic acid biosynthetic enzyme, AAO3, in Arabidopsis. Plant Physiology 134, 1697–1707. 15064376 10.1104/pp.103.036970 PMC419843

[CIT0018] Kumar MN Jane WN Verslues PE 2013 Role of the putative osmosensor *Arabidopsis* histidine kinase1 in dehydration avoidance and low-water-potential response. Plant Physiology 161, 942–953. 23184230 10.1104/pp.112.209791 PMC3561031

[CIT0019] Kushiro T Okamoto M Nakabayashi K Yamagishi K Kitamura S Asami T Hirai N Koshiba T Kamiya Y Nambara E 2004 The Arabidopsis cytochrome P450 CYP707A encodes ABA 8′-hydroxylases: key enzymes in ABA catabolism. The EMBO Journal 23, 1647–1656. 15044947 10.1038/sj.emboj.7600121 PMC391058

[CIT0020] Lee KH Piao HL Kim HY Choi SM Jiang F Hartung W Hwang I Kwak JM Lee IJ Hwang I 2006 Activation of glucosidase via stress-induced polymerization rapidly increases active pools of abscisic acid. Cell 126, 1109–1120. 16990135 10.1016/j.cell.2006.07.034

[CIT0021] McAdam SAM 2015 Physicochemical quantification of abscisic acid levels in plant tissues with an added internal standard by ultra-performance liquid chromatography. Bio-Protocol 5, 1–13.

[CIT0022] McAdam SAM Brodribb TJ 2015 The evolution of mechanisms driving the stomatal response to vapor pressure deficit. Plant Physiology 167, 833–843. 25637454 10.1104/pp.114.252940 PMC4348763

[CIT0023] McAdam SAM Brodribb TJ 2016 Linking turgor with ABA biosynthesis: implications for stomatal responses to vapor pressure deficit across land plants. Plant Physiology 171, 2008–2016. 27208264 10.1104/pp.16.00380 PMC4936570

[CIT0024] McAdam SAM Sussmilch FC Brodribb TJ 2016 Stomatal responses to vapour pressure deficit are regulated by high speed gene expression in angiosperms. Plant, Cell & Environment 39, 485–491. 10.1111/pce.12633 26353082

[CIT0025] Mott KA 1988 Do stomata respond to CO_2_ concentrations other than intercellular?Plant Physiology 86, 200–203. 16665866 10.1104/pp.86.1.200 PMC1054454

[CIT0026] Ng L-M Soon F-F Zhou XE 2011 Structural basis for basal activity and autoactivation of abscisic acid (ABA) signaling SnRK2 kinases. Proceedings of the National Academy of Sciences, USA 108, 21259–21264. 10.1073/pnas.1118651109 PMC3248506 22160701

[CIT0027] Okamoto M Tanaka Y Abrams SR Kamiya Y Seki M Nambara E 2009 High humidity induces abscisic acid 8′-hydroxylase in stomata and vasculature to regulate local and systemic abscisic acid responses in Arabidopsis. Plant Physiology 149, 825–834. 19036833 10.1104/pp.108.130823 PMC2633821

[CIT0028] Osakabe Y Osakabe K Shinozaki K Tran LS 2014 Response of plants to water stress. Frontiers in Plant Science 5, 86. 24659993 10.3389/fpls.2014.00086 PMC3952189

[CIT0029] Park SY Fung P Nishimura N 2009 Abscisic acid inhibits type 2C protein phosphatases via the PYR/PYL family of START proteins. Science 324, 1068–1071. 19407142 10.1126/science.1173041 PMC2827199

[CIT0030] Qin X Zeevaart JAD 1999 The 9-*cis*-epoxycarotenoid cleavage reaction is the key regulatory step of abscisic acid biosynthesis in water-stressed bean. Proceedings of the National Academy of Sciences, USA 96, 15354–15361. 10.1073/pnas.96.26.15354 PMC24823 10611388

[CIT0031] Raven JA 2002 Selection pressures on stomatal evolution. New Phytologist 153, 371–386. 10.1046/j.0028-646X.2001.00334.x 33863217

[CIT0032] Reiser V Raitt DC Saito H 2003 Yeast osmosensor Sln1 and plant cytokinin receptor Cre1 respond to changes in turgor pressure. The Journal of Cell Biology 161, 1035–1040. 12821642 10.1083/jcb.200301099 PMC2172993

[CIT0033] Saito S Hirai N Matsumoto C Ohigashi H Ohta D Sakata K Mizutani M 2004 Arabidopsis CYP707As encode (+)-abscisic acid 8′-hydroxylase, a key enzyme in the oxidative catabolism of abscisic acid. Plant Physiology 134, 1439–1449. 15064374 10.1104/pp.103.037614 PMC419820

[CIT0034] Schmittgen TD Livak KJ 2008 Analyzing real-time PCR data by the comparative *C*_T_ method. Nature Protocols 3, 1101–1108. 18546601 10.1038/nprot.2008.73

[CIT0035] Shankarnarayan S Narang SS Malone CL Deschenes RJ Fassler JS 2008 Modulation of yeast Sln1 kinase activity by the CCW12 cell wall protein. The Journal of Biological Chemistry 283, 1962–1973. 18048366 10.1074/jbc.M706877200 PMC2892218

[CIT0036] Soon FF Ng LM Zhou XE 2012 Molecular mimicry regulates ABA signaling by SnRK2 kinases and PP2C phosphatases. Science 335, 85–88. 22116026 10.1126/science.1215106 PMC3584687

[CIT0037] Thompson AJ Jackson AC Symonds RC Mulholland BJ Dadswell AR Blake PS Burbidge A Taylor IB 2000 Ectopic expression of a tomato 9-*cis*-epoxycarotenoid dioxygenase gene causes over-production of abscisic acid. The Plant Journal 23, 363–374. 10929129 10.1046/j.1365-313x.2000.00789.x

[CIT0038] Tran L-SP Urao T Qin F Maruyama K Kakimoto T Shinozaki K Yamaguchi-Shinozaki K 2007 Functional analysis of AHK1/ATHK1 and cytokinin receptor histidine kinases in response to abscisic acid, drought, and salt stress in Arabidopsis. Proceedings of the National Academy of Sciences, USA 104, 20623–20628. 10.1073/pnas.0706547105 PMC2154481 18077346

[CIT0039] Umezawa T Okamoto M Kushiro T Nambara E Oono Y Seki M Kobayashi M Koshiba T Kamiya Y Shinozaki K 2006 CYP707A3, a major ABA 8′-hydroxylase involved in dehydration and rehydration response in *Arabidopsis thaliana*. The Plant Journal 46, 171–182. 16623881 10.1111/j.1365-313X.2006.02683.x

[CIT0040] Urao T Yakubov B Satoh R Yamaguchi-Shinozaki K Seki M Hirayama T Shinozaki K 1999 A transmembrane hybrid-type histidine kinase in Arabidopsis functions as an osmosensor. The Plant Cell 11, 1743–1754. 10488240 10.1105/tpc.11.9.1743 PMC144312

[CIT0041] Wohlbach DJ Quirino BF Sussman MR 2008 Analysis of the *Arabidopsis* histidine kinase ATHK1 reveals a connection between vegetative osmotic stress sensing and seed maturation. The Plant Cell 20, 1101–1117. 18441212 10.1105/tpc.107.055871 PMC2390728

[CIT0042] Xie X Wang Y Williamson L 2006 The identification of genes involved in the stomatal response to reduced atmospheric relative humidity. Current Biology 16, 882–887. 16682349 10.1016/j.cub.2006.03.028

[CIT0043] Zhang YJ Rockwell FE Graham AC Alexander T Holbrook NM 2016 Reversible leaf xylem collapse: a potential ‘Circuit Breaker’ against cavitation. Plant Physiology 172, 2261–2274. 27733514 10.1104/pp.16.01191 PMC5129713

[CIT0044] Zhao M Running SW 2010 Drought-induced reduction in global terrestrial net primary production from 2000 through 2009. Science 329, 940–943. 20724633 10.1126/science.1192666

