# Supplementary material for: Up-regulation of NCED3 and ABA biosynthesis occur within minutes of a decrease in leaf turgor but AHK1 is not required
Source: J Exp Bot. 2017 Apr 25;68(11):2913–8. doi: 10.1093/jxb/erx124 (PMC5853609; doi:10.1093/jxb/erx124)
Supplement: supplementary_figures_S1_S4 [file erx124_suppl_supplementary_figures_s1_s4.pdf]

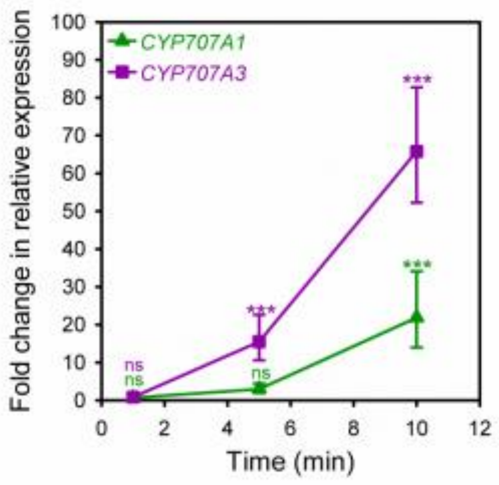


**Supplementary Figure S1:** Expression of ABA catabolism genes.

Fold change in relative gene expression in wild-type *Arabidopsis* leaves after exposure to external pressure of 1.5 MPa (at 0 min), relative to initial levels. Error bars indicate 95% CI for n = 3 biological replicates. The significance of differences between $\Delta C_T$ values (gene of interest relative to reference gene *MON1*) for samples from each timepoint compared with initial samples, as determined by ANOVA and Dunnet's 2-sided t-test, is indicated: ***, $P \leq 0.001$; ns, not significant.

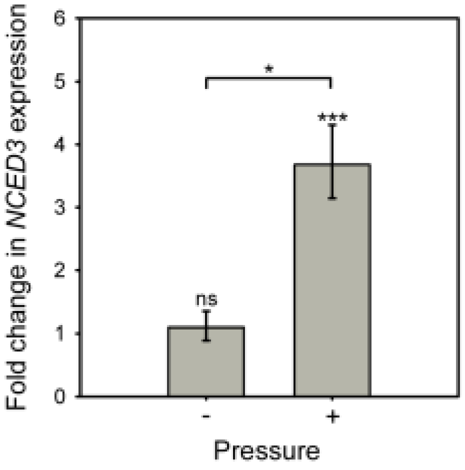


**Supplementary Figure S2:** Confirmation that *NCED3* upregulation is triggered by application of external pressure.

Fold change in *NCED3* expression in wild-type *Arabidopsis* leaves after 20 minutes in the pressure chamber without (-) and with (+) application of external pressure of 1.5 MPa, relative to initial levels. Error bars indicate 95% CI for n = 3-4 biological replicates. The significance of differences between $\Delta C_T$ values (*NCED3* relative to reference gene *MON1*) for initial samples compared with samples from final time-points, and between $\Delta\Delta C_T$ values, as determined by t-test, is indicated: *, $P \leq 0.05$; ***, $P \leq 0.001$; ns, not significant. Data for samples exposed to pressure is also shown in Fig. 2A.

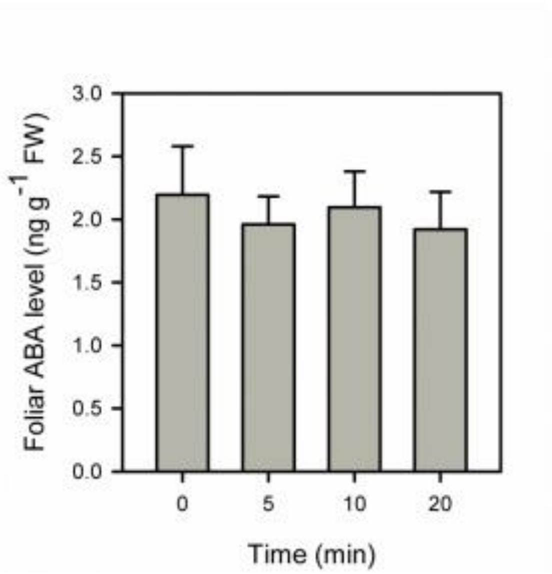


**Supplementary Figure S3:** Foliar ABA level does not change in leaves which are not exposed to external pressure.

Foliar ABA level in wild-type *Arabidopsis* leaves after containment in a Scholander pressure chamber under no external pressure. Error bars indicate SE for n = 3 biological replicates: P=0.911 by ANOVA.

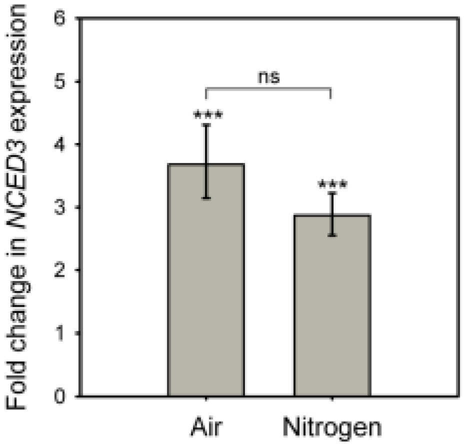


**Supplementary Figure S4:** Confirmation that *NCED3* upregulation is triggered by changes to turgor pressure and not altered $CO_2$ levels when external pressure is applied.

Fold change in *NCED3* expression in wild-type *Arabidopsis* leaves after exposure to external pressure of 1.5 MPa relative to initial levels, under air (which could potentially alter $CO_2$ levels) and nitrogen gas. Error bars indicate 95% CI for n = 3-4 biological replicates. Stars denote significant differences between $\Delta C_T$ values (*NCED3* relative to reference gene *MON1*) for initial samples compared with samples from final time-points, as determined by t-test: ***, $P \leq 0.001$. A t-test confirmed no significant difference (ns) between $\Delta\Delta C_T$ values when performed under air and nitrogen gas ($P = 0.324$), showing that there is a comparable increase in *NCED3* expression induced by altered turgor under both air and nitrogen gas. Data for air is also shown in **Fig. 2A**.
